# Quinoline Derivative Enhances Human Sperm Motility and Improves the Functional Competence

**DOI:** 10.1007/s43032-020-00382-5

**Published:** 2020-11-25

**Authors:** Sandhya Kumari, Sujith Raj Salian, Arpitha Rao, Shilpa M. Somagond, Ravindra R. Kamble, Aravind Nesaragi, Jyotirekha Das, G. K. Rajanikant, Srinivas Mutalik, Shamprasad Varija Raghu, Satish Kumar Adiga, Guruprasad Kalthur

**Affiliations:** 1 Department of Clinical Embryology, Kasturba Medical College, Manipal, Manipal Academy of Higher Education, Manipal, Karnataka 576 104 India; 2 Department of Studies in Chemistry, Karnatak University, Dharwad, Karnataka 580 003 India; 3 School of Biotechnology, National Institute of Technology Calicut, Calicut, Kerala 673 601 India; 4 Department of Pharmaceutics, Manipal College of Pharmaceutical Sciences, Manipal Academy of Higher Education, Manipal, Karnataka 576 104 India; 5 Neurogenetics Lab, Department of Applied Zoology, Mangalore University, Mangalaganothri, Konaje, Karnataka 574199 India

**Keywords:** Quinoline derivative, Phosphodiesterase inhibitors, Sperm motility, Cyclic AMP, Tyrosine phosphorylation of sperm protein, Fertilization

## Abstract

In this study, we aimed to explore the beneficial properties of novel quinoline derivatives on human sperm motility and its functional competence. Nine novel quinoline derivatives were screened for their effect on motility in human spermatozoa from normozoospermic ejaculates. Compounds with impressive sperm motility enhancement properties were further assessed for their effect on functional competence of human spermatozoa. To determine the effect on the fertilizing ability of spermatozoa processed with quinoline derivatives and to assess developmental competence of embryos derived, in vitro fertilization (IVF) was performed using mouse model. Among the nine quinoline derivatives, 2 compounds (6MQT and 2,6DQT) exhibited significant enhancement in sperm progressive motility and survival at 24 h. Further, non-significant increase in curvilinear velocity (VCL), straight line velocity (VSL), and amplitude of lateral head displacement (ALH) was observed. Capacitation, intracellular cAMP level and tyrosine phosphorylated sperm proteins were significantly higher in 6MQT (*P* < 0.05) and 2,6DQT (*P* < 0.001) compared to control. In vitro fertilization (IVF) experiments using Swiss albino mice revealed that spermatozoa processed with 6MQT had non-significantly higher blastocyst rate and a superior blastocyst quality, while, 2,6DQT resulted in significantly lower blastocyst rate (*P* < 0.05) compared to control. Quinoline derivative 6MQT has significant motility enhancement property under in vitro conditions.

**Supplementary Information:**

The online version contains supplementary material available at 10.1007/s43032-020-00382-5.

**Figure:**
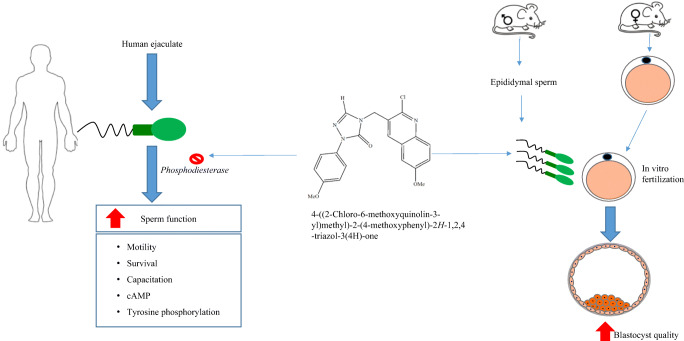
Graphical abstract

## Introduction

Motility is an important physiological attribute of spermatozoa, which is essential for successful fertilization in both natural and artificial conception methods. The percentage of motile spermatozoa present in the ejaculated semen sample helps the fertility specialist to determine the most appropriate infertility treatment method. In medically assisted conception methods such as intrauterine insemination (IUI), in vitro fertilization (IVF), and intracytoplasmic sperm injection (ICSI), extraction of motile spermatozoa from the neat ejaculate is an important step. Total motile spermatozoa count (TMSC) is considered to be the strongest predictive marker for IUI pregnancy and can act as an indicator of severity of male factor infertility [1, 2]. Further, motility enables the quick selection of viable and most appropriate spermatozoa for microinjection procedures during ICSI [3]. Therefore, poor motility not only reduces the likelihood of fertilization during natural conception, but can also cause certain technical difficulties during ICSI, since the selection of viable sperm for microinjection depends on motility. Consequently, agents that improve sperm motility in vitro may have significant therapeutic implications for infertility treatment [4, 5].

Poor understanding of the regulatory mechanism of motility poses challenges in developing novel sperm motility enhancers [6]. Pharmacological agents such as pentoxifylline, caffeine, theophylline, theobromine, and 2-deoxyadenosine, which have inhibitory effect on phosphodiesterases, have demonstrated sperm motility enhancement [7, 8]. However, due to the contradictory reports on their beneficial role in sperm motility enhancement, they are not used routinely in infertility treatment [9–12]. Few studies have shown adverse effects of pentoxifylline on gametes and embryo development [13–15]. Therefore, the identification or synthesis of a molecule that enhances sperm motility without having any adverse effects on the oocyte or embryo development has a potential therapeutic utility in the treatment of infertility.

**Fig. 1:**
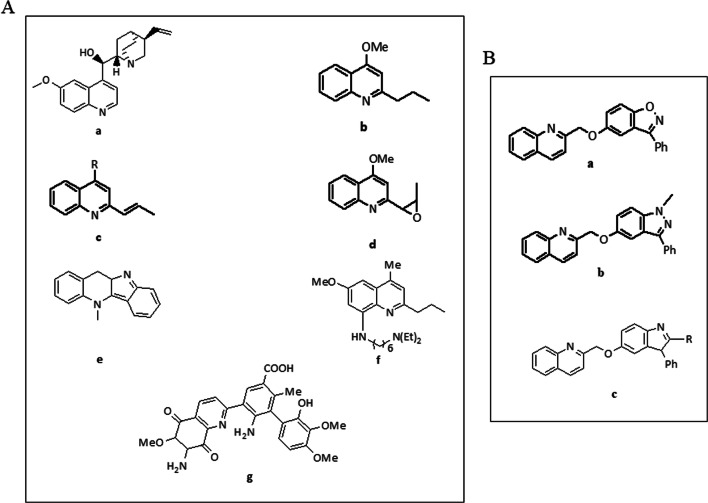
(A) Scaffolds containing (a) Quinine; (b) 4-Methoxy-2-propylquinoline; (c) 4-Substituted-2-((E)-prop-1-enyl)quinoline; (d) 4-Methoxy-2-(3-methyloxiran-2-yl)quinoline; (e) 10a,11-Dihydro-5-methyl-5H-indolo[3,2-b]quinoline; (f) 8-(6-N,N-Dethylhexyl)amino-6-methoxy-4-methyl-2-propylaylquinoline; and (g) 2-Biaryl-6-methoxy-7-amino-5,8-dioxo-quinoline. (B) Scaffolds containing (a) 2-((3-Phenylbenzo[d]isoxazol-5-yloxy)methyl)quinoline; (b) 2-((1-Methyl-3-phenyl-1H-indazol-5-yloxy)methyl)quinoline; and (c) 2-((2-Sustituted-3-phenyl-3H-indol-5-yloxy)methyl)quinoline

Quinoline is the most important benzfused nitrogen containing aromatic compound. Quinoline derivatives have received the attention of researchers due to their broad range of pharmacological activities [16]. The common sources of quinoline are petroleum, coal processing, preservation of wood, and shale oil [17]. Quinine, which is used in the treatment of malaria, has been isolated from the bark of the *Cinchona calisaya* tree [18]. Natural quinoline derivatives (Fig. 1(A)) have also been obtained from the different plants [19, 20]. Quinoline derivatives viz., pamaquine, chloroquine, tafenoquine, bulaquine, and mefloquine which are of synthetic origin are used as antimalarial and anti-inflammatory agents [21–24]. Structural analogs of compounds containing benzoxazole, phenylindazole, and indole rings attached to quinoline through oxygen as linker group (Fig. 1(B)) are shown to possess inhibitory effect on phosphodiesterases (PDEs) [25]. Since inhibition of PDEs in spermatozoa can enhance the motility, we were interested in assessing whether the novel quinoline derivatives synthesized by our group [26] have any significant beneficial effect on motility and other physiological properties in human spermatozoa.

## Materials and Methods

### Preparation of Quinoline Derivatives

Nine quinoline derivatives were synthesized according to the procedure reported elsewhere [26]. The IUPAC naming, molecular weight, and the codes for these compounds are given in Table 1. Stock solution (1 mg/mL) of these compounds were prepared by dissolving in dimethyl sulfoxide (DMSO). Working solutions were prepared freshly each time in EBSS (Earl’s balanced salt solution, Sigma-Aldrich, Cat. No. E2888) media containing 0.1% bovine serum albumin (BSA, Sigma-Aldrich, Cat. No. A3311).

**Table 1:**
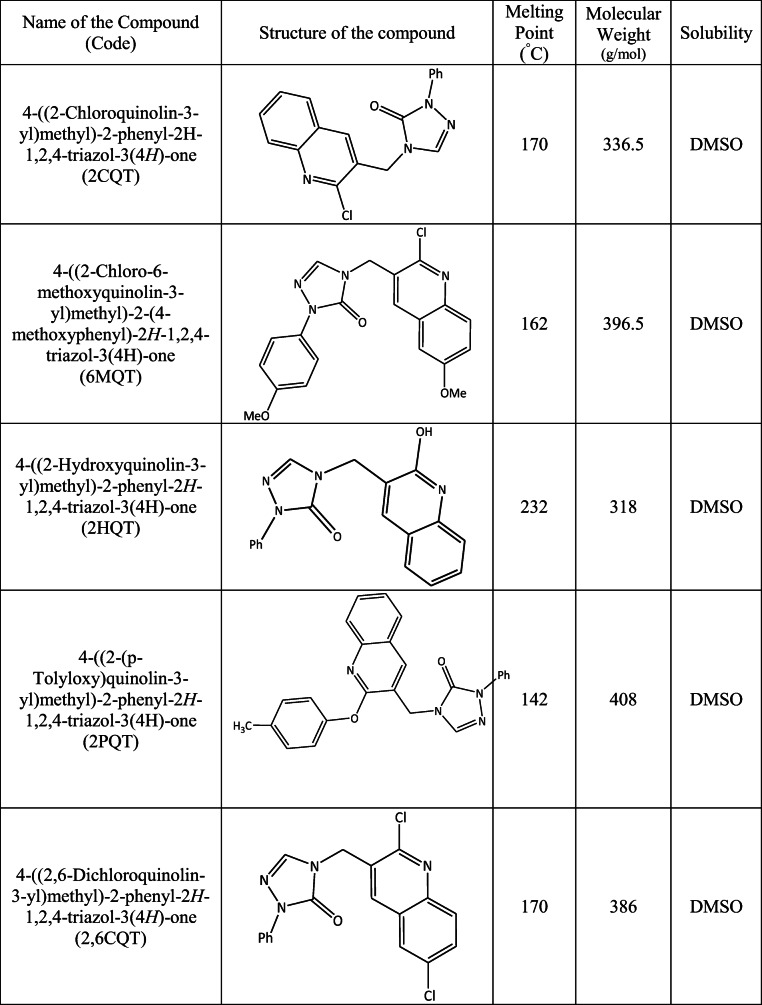

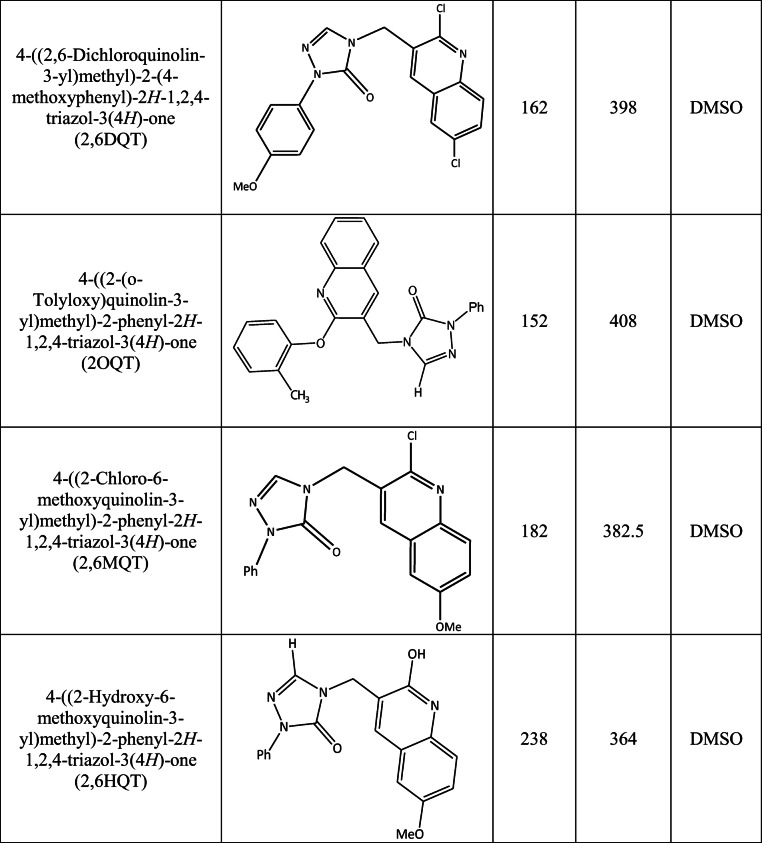
Synthesized quinoline derivatives with details of their IUPAC name, code, structure, melting point, molecular weight, and solubility

### Study Subjects

Normozoospermic infertile men (*n* = 73) attending Andrology laboratory for routine semen analysis were included in the study (Supplementary information Table 1). A written consent was obtained from subjects willing to provide semen samples. A prior approval was granted for the study by the Institutional Ethics committee of Kasturba Medical College, Manipal, Manipal Academy of Higher Education, Manipal (IEC 155/2015).

### Animals for In Vitro Fertilization

Swiss albino mice (6–8 weeks) obtained from Central Animal Facility, Kasturba Medical College, Manipal, Manipal Academy of Higher Education, Manipal, were used for the experiments. Animals were maintained under standard conditions of light (12:12 h of light and dark), temperature (23 ± 2 °C), and food and water ad libitum. A prior approval was obtained for this study from the Institutional Animal Ethical Committee (IAEC/KMC/62/2016).

### Study Outline for Screening of Quinoline Derivatives for Sperm Motility Enhancement

After routine semen analysis, the leftover normozoospermic samples were used for screening the effect of 9 newly synthesized quinoline derivatives on sperm motility under in vitro conditions. Among the 9 quinoline derivatives screened, 2 compounds, viz- 6MQT ((4-((2-Chloro-6-methoxyquinolin-3-yl)methyl)-2-(4-methoxyphenyl)-2*H*-1,2,4-triazol-3(4*H*)-one) and 2,6DQT ((4-((2,6-Dichloroquinolin-3-yl)methyl)-2-(4-methoxyphenyl)-2*H*-1,2,4-triazol-3(4*H*)-one) showed considerable increase in total and progressive motility compared to control and other quinoline derivatives (Supplementary Table 2). The optimum concentration for 6MQT and 2,6DQT was found to be 0.05 μg/mL and 0.025 μg/mL respectively (Supplementary Tables 3 and 4). Therefore, for further studies to assess the sperm functional characteristics, these two concentrations were used.

### Effect of Quinoline Derivatives (6MQT and 2,6DQT) on Sperm Functional Characteristics

Equal volume of liquefied semen samples was washed with EBSS medium by centrifuging two times [27]. Sperm pellet obtained after centrifugation was resuspended in EBSS medium, divided into 4 groups, and incubated with EBSS media containing 0.1% BSA at 37 °C and 5% CO_2_ as mentioned below.

a. Control (C): incubated with EBSS media.

b. Vehicle control (VC): Incubated with EBSS containing 0.01% of DMSO.

c. 6MQT: Incubated with EBSS containing 0.05 μg/mL of 6MQT.

d. 2,6DQT: Incubated with EBSS containing 0.025 μg/mL of 2,6DQT.

After 1 h of incubation, the supernatant containing motile sperm fraction was collected without disturbing the pellet and used for further analysis.

### Sperm Cryopreservation and Thawing

The frozen semen samples (*n* = 20) donated for research from patients undergoing IVF at the university infertility clinic were used. The samples were cryopreserved by mixing with sperm freeze medium (SpermFreeze Medium-SFM, FertiPro, Belgium, Cat. No. FP14SF18). The aliquots were transferred to screw-top cryovials (Nunc, Denmark, Cat. No. V76340) and stored in liquid nitrogen (− 196 °C) until further use for the experiment. Frozen samples were thawed and cryoprotectants were removed by washing in EBSS medium [28]. The pellet obtained was overlaid with EBSS culture media supplemented with optimum concentration of 6MQT or 2,6DQT. After 1 h incubation at 37 °C and 5% CO_2_, the motile sperm fraction was carefully collected without disturbing the pellet and further incubated up to 24 h at 37 °C and 5% CO_2_.

### Motility and Kinematic Changes in Spermatozoa

For motility assessment, 10 μL of sperm suspension was placed on a microscopic slide and covered with a coverslip. Based on the motility pattern, spermatozoa were categorized into progressive, non-progressive, and immotile spermatozoa in 200 spermatozoa by observing under the light microscope (× 400 magnification). Kinematic changes in spermatozoa were assessed using CASA (computer-assisted sperm analysis). Approximately 10 μL of sperm suspension was placed on a clean microscopic slide and covered with coverslip. The slides were observed under trinocular microscope (UB200i, × 100 magnification, phase contrast objective). Straight line velocity (VSL), curvilinear velocity (VCL), amplitude of lateral head displacement (ALH), and average path velocity (VAP) were assessed in at least ten random field per sample [27] by using ISAS software (Prosier, Spain).

### Sperm Mitochondrial Membrane Potential

Mitochondrial membrane potential in spermatozoa was assessed at 1 h after incubation. Motile spermatozoa obtained from various groups was incubated with Rhodamine 123 (Sigma-Aldrich, USA, Cat. No. R8004) for 20 min. The pellet obtained by centrifugation at 1800 rpm for 8 min was resuspended in EBSS media. Ten microliters of sperm suspension was placed on clean microscopic slide and observed under fluorescence microscope (Image A1, Carl Zeiss, Germany). Minimum of 500 spermatozoa were scored from each group. Spermatozoa having bright fluorescence at the midpiece region were considered to have functional mitochondria. The percentage of spermatozoa with functional mitochondria was calculated and expressed in percentage [28].

### Sperm Capacitation by Chlortetracycline Assay

Sperm suspension obtained after swim-up from 4 groups was assessed for their ability to undergo capacitation by chlortetracycline (CTC) assay as described by Kotdawala et al. [28]. Briefly, 18 μL of sperm suspension was incubated with 2 μL ethidium bromide (EB, 23.3 μM) and 20 μL of CTC solution (750 μM) at 37 °C in the dark. After 15 s, 5 μL of glutaraldehyde solution was added and capacitation pattern was evaluated using fluorescence microscope. From each sample, 500 spermatozoa were scored and classified as capacitated, non-capacitated, and dead spermatozoa. Based on the staining pattern, spermatozoa were classified as un-capacitated-bright fluorescence over the entire sperm head and midpiece region; capacitated spermatozoa, fluorescence in the anterior portion of the head; acrosome reacted, low fluorescence throughout the sperm head, with positive signal in the midpiece; and dead spermatozoa, red fluorescence in the head region of spermatozoa. Capacitated and acrosome reacted spermatozoa were counted and the results are expressed in percentage of capacitated spermatozoa.

### Calcium Ionophore-Induced Acrosome Reaction Assay

The fertilizing ability of the spermatozoa was assessed using a method described earlier [27]. Briefly, the motile sperm fraction obtained from various groups was incubated with or without calcium ionophore A23187 (5 μM, Sigma-Aldrich, Cat. No. C7522) at 37 °C for 1 h. Following incubation, sperm suspension was washed with phosphate buffered solution (PBS) and smeared on a coverslip. The cells were fixed with methanol and stained with FITC-conjugated *Pisum sativum* agglutinin (FITC-PSA, Sigma-Aldrich, Cat. No. L0770), followed by wash with Milli-Q water, counterstained with propidium iodide (PI, Sigma-Aldrich, Cat. No. P4170), and then mounted on slide using mounting media (DAKO, Cat. No. S3023). Percentage of acrosome reacted spermatozoa (without green florescence on acrosome region) were calculated by counting 500 spermatozoa by observing under fluorescence microscope.

### Assessment of Sperm DNA Damage by Terminal Deoxynucleotidyl Transferase (TdT) dUTP Nick End Labelling Assay

The percentage of DNA damaged spermatozoa were evaluted by terminal deoxynucleotidyl transferase (TdT) dUTP nick end labelling (TUNEL) assay using kit from Rosche diagnosticis (Cat. No. 12156792910) as described earlier [29]. Briefly, sperm suspension incubated with or without 6MQT and 2,6DQT for 24 h were fixed on coverslips with 4% paraformaldehyde (PFA) solution, washed with phosphate buffered saline (PBS), and then permeablized for 1 h. Samples were then washed with PBS and incubated with TUNEL reaction mixture for 1 h in dark. Then samples were washed with PBS again, counter stained with DAPI (4′,6-diamidino-2-phynylindole), and fixed on clean glass slide using mounting medium. Slides were observed under fluoroscence microscope and sperm DNA damage was assessed in minimum of 500 spermatozoa and results were expressed in percentage.

### Detection of cAMP Level in Spermatozoa

Level of intracellular cAMP in spermatozoa was measured using cAMP test kit according to the manufacturer’s protocol (Elabscience®, Cat. No. E-EL-0056). The sperm concentration was adjusted to 3 million/mL in each group. The samples were sonicated for 30 s using sonicator (Sonics Vibra-Cell VCX130 Ultrasonic, Amplitude 30%) and centrifuged at 3000 rpm for 10 min. Supernatant was collected and stored at − 80 °C until further analysis. For cAMP detection, 50 μL standard or sonicated samples were added to each well. Immediately, 50 μL biotinylated detection Ab was added and incubated for 45 min at 37 °C. The solution from each well was decanted and washed 3 times using wash buffer followed by adding 100 μL horseradish peroxidase (HRP) conjugate to each well. After 30 min incubation at 37 °C, each well was washed repeatedly (5 times) followed by addition of 90 μL substrate reagent and incubated for 15 min at 37 °C. Further, the reaction was stopped by adding 50 μL of stop solution and immediately optical density (OD) of the each well was measured at 450 nm using Skanit software 5.0 (Multiskan, Photometer, Thermoscientific). The assay was performed in duplicates. A standard curve was drawn, and the concentration was calculated from the curve [27].

### Assesment of Tyrosine Phosphorylation of Sperm Protiens by Immunofluorescence

Motile spermatozoa collected by swim-up technique were incubated for 1 h at 37 °C and 5% CO_2_. The spermatozoa were fixed on coverslip using 4% (w/v) paraformaldehyde in PBS. Permeabilization of fixed spermatozoa was done by using 0.1% (v/v) Triton X-100 in PBS for 10 min at room temperature followed by 2 washes in PBS, 5 min each. The spermatozoa were blocked by incubating with 1% BSA in PBS containing 0.1% Tween-20 for 1 h followed by incubation at 4 °C overnight with anti phospho-tyrosine antibody (1:100, Clone PY20, Merck Millipore, Cat. No. 05-947). After washing, the spermatozoa were incubated with antimouse IgG-FITC (1:1000, Santa Cruz, USA, Cat. No. S6MQT0101), cells were counterstained with DAPI and observed under flourescence microscope. The number of tyrosine phosphoprotien postive spermatozoa was counted in total of 500 spermatozoa and the results were expressed in percentage.

### Effect of 6MQT and 2,6DQT Supplementation to Sperm Wash Medium on Fertilization and Preimplantaion Embryo Development in Swiss Albino Mice

The effect of 6MQT and 2,6DQT on fertilization and subsequent preimplantaion embryo development was assessed in murine model. Briefly, the caudal spermatozoa from Swiss albino mice were collected in EBSS media containing 0.1% BSA. After 2 h of incubation, the sperm suspension was centrifuged at 1800 rpm for 8 min and pellet obtained was divided into four parts—control (C), vehicle control (VC, 0.01% DMSO), 6MQT, and 2,6DQT. Motile spermatozoa were extracted by swim-up technique by carefully layering the sperm pellet with EBSS media containing 2.5% BSA (C) with or without 6MQT and 2,6DQT for 1 h. The motile spermatozoa collected from C, VC, 6MQT, and 2,6DQT were used for in vitro fertilization. Oocyte cumulus complexes (OCC’s) collected from the oviduct of superovulated female mice were divided into 4 groups and inseminated with sperm suspension from C, VC, 6MQT, and 2,6DQT as explained previously [27]. The fertilized oocytes were then washed in M16 media and cultured till blastocyst stage to assess the blastocyst and hatching rate. DNA damage in blastocysts was assessed by TUNEL assay [30].

### In Silico Binding Affinity Studies of 6MQT and 2,6DQT with Various Isoforms of Phosphodiesterase’s

Molecular docking predicts the preferred orientation of a small molecule to its target protein when bound together to form a stable complex. The ligands (6MQT and 2,6DQT), reported small molecule inhibitors and pentoxifylline (non-specific phosphodiesterase (PDE) inhibitor) were computationally docked into the predicted binding pockets on the proteins to build protein-ligand complex structures [31]. A confidence score (c-score) in the range of [0–1] was given to determine the accuracy of each prediction, where a higher score implies a more accurate prediction. The docking energies for complex structures were shown in the “Energy” columns. Further, the amino acid residues involved in the interactions with the ligand were also listed.

### Statistical Evaluvation

All the data were represented as mean and standard error (mean ± SEM), except embryo developmental paramers which are given as percentage data. One way analysis of varience (ANOVA) test was used to compare the difference among the groups and percentage data was analyzed by chi-square test using GraphPad InSata 3.0 stastical package (GraphPad Inc., USA). *P* value < 0.05 was considered stastically significant. All the graphs were prepared using Microcal Origin 6.0 (Origin Lab Corporation, Northampton, MA, USA).

## Results

### Effect of Quinoline Derivatives on Sperm Motility and Sperm Kinematics

**Fig. 2:**
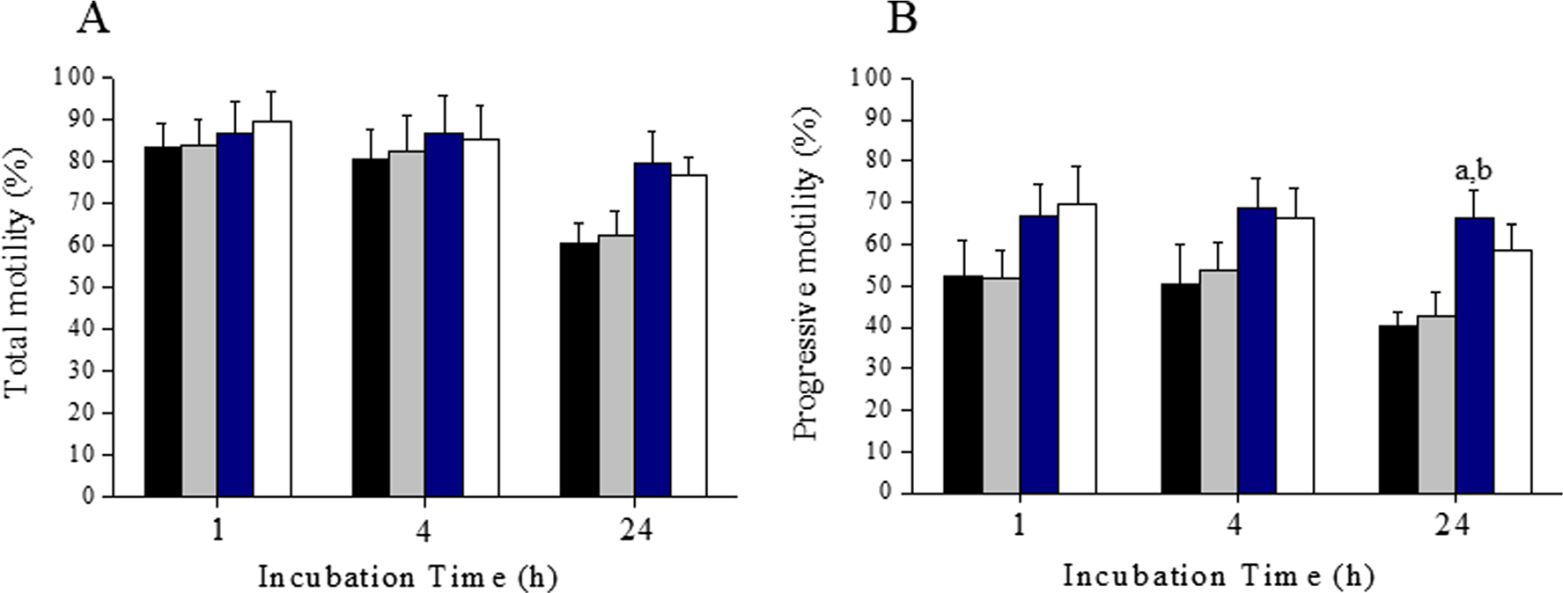
Effect of 6MQT (0.05 μg/mL) and 2,6DQT (0.025 μg/mL) on (A) total and, (B) progressive sperm motility in ejaculated spermatozoa (*n* = 11) at various time intervals (1, 4, and 24 h) after processing by swim-up technique. Data represents mean ± SEM. ^a^*P* < 0.05 v/s C; ^b^*P* < 0.05 v/s VC. Control (C) (black bar); Vehicle control (VC) (gray bar); 6MQT (blue bar); 2,6DQT (white bar)

In fresh ejaculates, presence of 6MQT and 2,6DQT in sperm wash medium did not result in any significant increase in total motility at 1 and 4 h incubation time (Fig. 2(A)). Similarly, the percentage of spermatozoa with progressive motility was non-significantly higher in 6MQT and 2,6DQT group at these two intervals (Fig. 2(B)). However, at 24 h after incubation, 6MQT group had significantly higher progressive motility compared to control (*P* < 0.05).

**Fig. 3:**
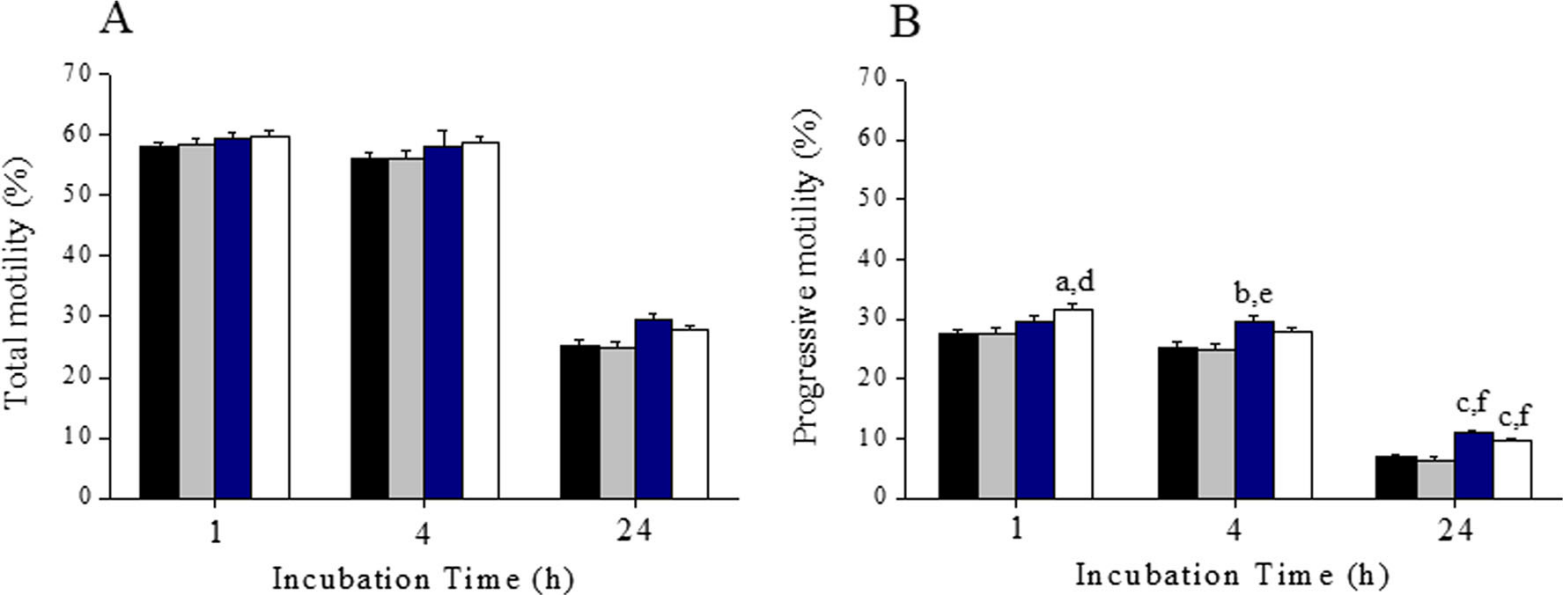
Effect of 6MQT (0.05 μg/mL) and 2,6DQT (0.025 μg/mL) on (A) total and, (B) progressive sperm motility in frozen-thawed (*n* = 20) human spermatozoa at various time intervals (1, 4, and 24 h) after processing by swim-up technique. Data represents mean ± SEM. ^a^*P* < 0.05, ^b^*P* < 0.01, ^c^*P* < 0.001 v/s C; ^d^*P* < 0.05, ^e^*P* < 0.01, ^f^*P* < 0.001 v/s VC. Control (C) (black bar); Vehicle control (VC) (gray bar); 6MQT (blue bar); 2,6DQT (white bar)

**Fig. 4:**
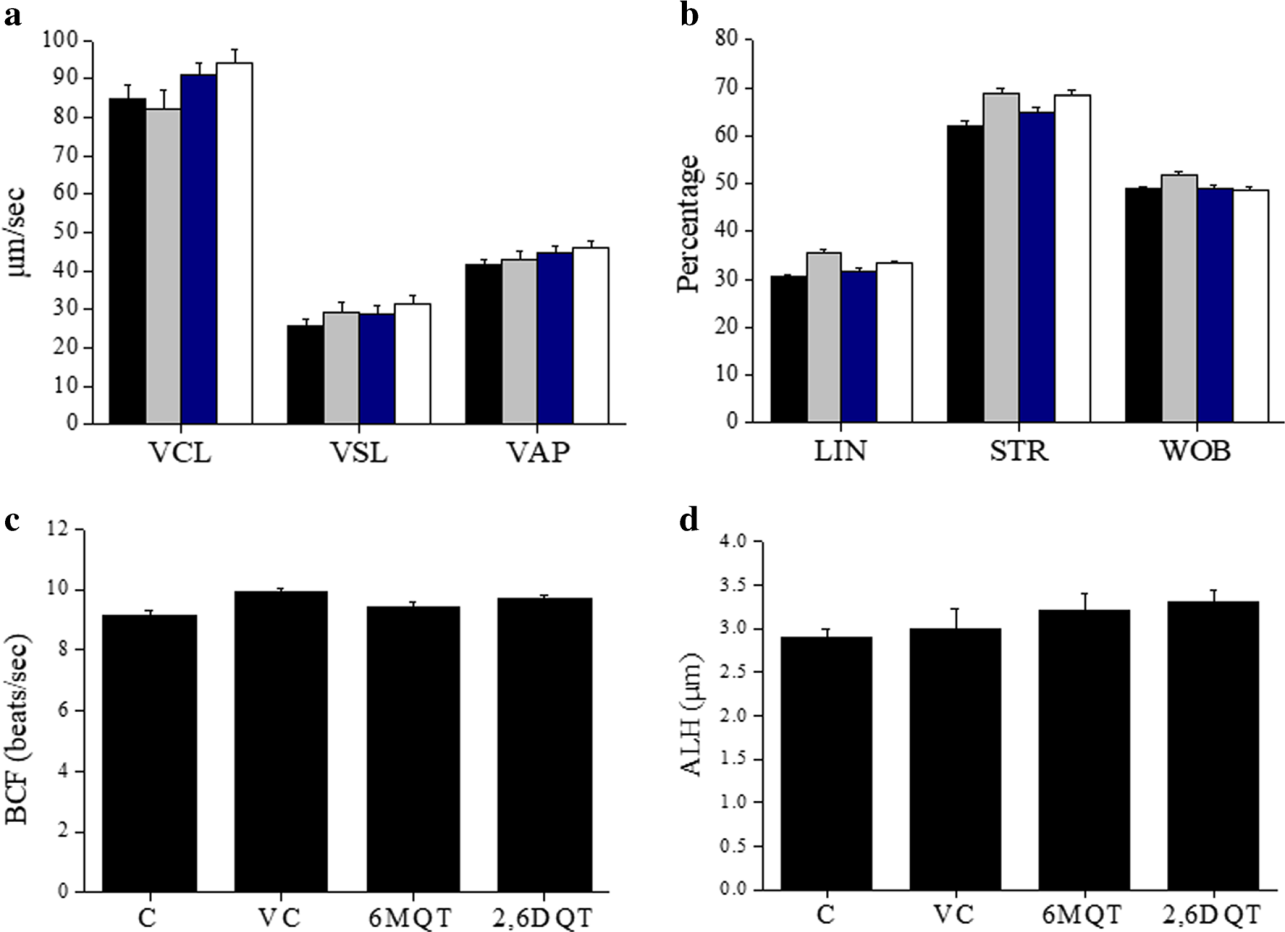
Effect 6MQT (0.05 μg/mL) and 2,6DQT (0.025 μg/mL) on sperm kinematics assessed using computer-assisted sperm analysis (CASA) in motile sperm fraction collected by swim-up at 1 h after incubation in vitro (*n* = 10). (A) VCL, VSL, and VAP; (B) LIN, STR, WOB; C) BCF; D) ALH. Data represents mean ± SEM. Control (C) (black bar); Vehicle control (VC) (gray bar); 6MQT (blue bar); 2,6DQT (white bar)

Further, we wanted to test whether the motility enhancement is observed even in frozen-thawed samples. As observed with fresh ejaculates, at 1 and 4 h interval, there was no significant difference in the total motility in 6MQT and 2,6DQT groups (Fig. 3(A)). However, progressive motility was significantly higher in 2,6DQT group at 1 h (*P* < 0.05) and in 6MQT group at 4 h (*P* < 0.01) (Fig. 3(B)). At 24 h interval, both the quinoline derivatives had significantly higher total and progressive motility (*P* < 0.01). When the kinematics of spermatozoa from fresh ejaculates processed with 6MQT and 2,6DQT was assessed at 1 h after incubation, no significant differences in VCL, VSL, VAP, ALH, LIN, STR, and BCF were observed (Fig. 4(A–D)).

### Assessment of Mitochondrial Membrane Potential and DNA Integrity

**Fig. 5:**
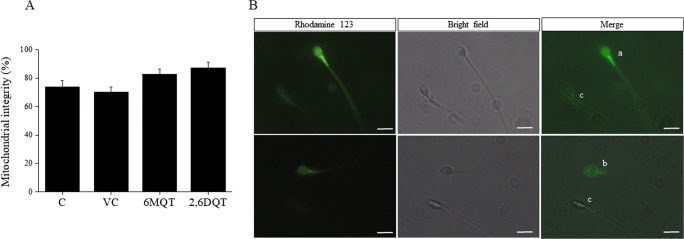
(A) Effect of 6MQT (0.05 μg/mL) and 2,6DQT (0.025 μg/mL) on mitochondrial integrity in motile sperm fraction collected by swim-up at 1 h after incubation in vitro; Data represents mean ± SEM (*n* = 18). (B) Representative images of spermatozoa stained with Rhodamine 123. (a) Spermatozoa with intact mitochondrial potential (brightly stained midpiece region); (b) spermatozoa with partial mitochondrial damage (partially stained smidpiece region); and (c) spermatozoa with damaged mitochondrial potential (unstained midpiece region), Magnification × 1000. Scale bar represents 10 μm

**Fig. 6:**
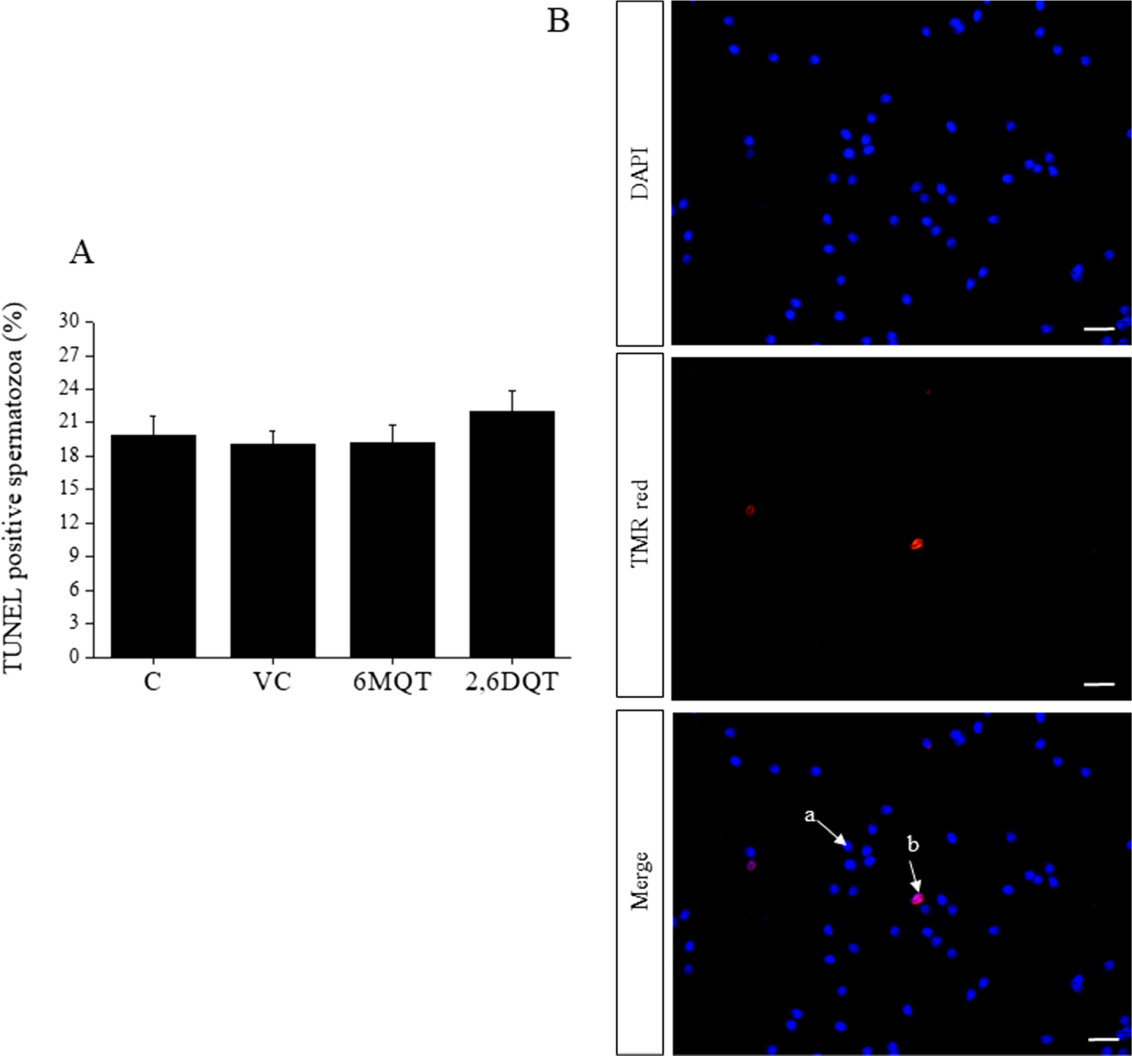
(A) Effect of 6MQT (0.05 μg/mL) and 2,6DQT (0.025 μg/mL) on, sperm DNA damage assessed by TUNEL assay in motile sperm fraction collected by swim-up at 24 h after incubation in vitro*.* Data represents mean ± SEM (*n* = 18). (B) Representative images of (a) spermatozoa with intact DNA; and (b) spermatozoa with DNA damage (TMR red positive). x1000 magnification. Scale bar represents 10 μm

At 1 h after incubation, the percentage of spermatozoa with intact mitochondrial membrane potential was non-significantly higher in 6MQT and 2,6DQT compared to control (Fig. 5) indicating that they do not have any adverse effects on the sperm mitochondria function, which also correlates with the motility and kinematics data. Further, with the incubation time up to 24 h in vitro*,* no significant difference was observed among the groups (data not shown). Similarly, as evident from the TUNEL assay results, incubation of spermatozoa in medium supplemented with 6MQT and 2,6DQT did not affect the sperm DNA integrity (Fig. 6).

### Assessment of Fertilizing Ability of Spermatozoa by Capacitation and Acrosome Reaction Assay

**Fig. 7:**
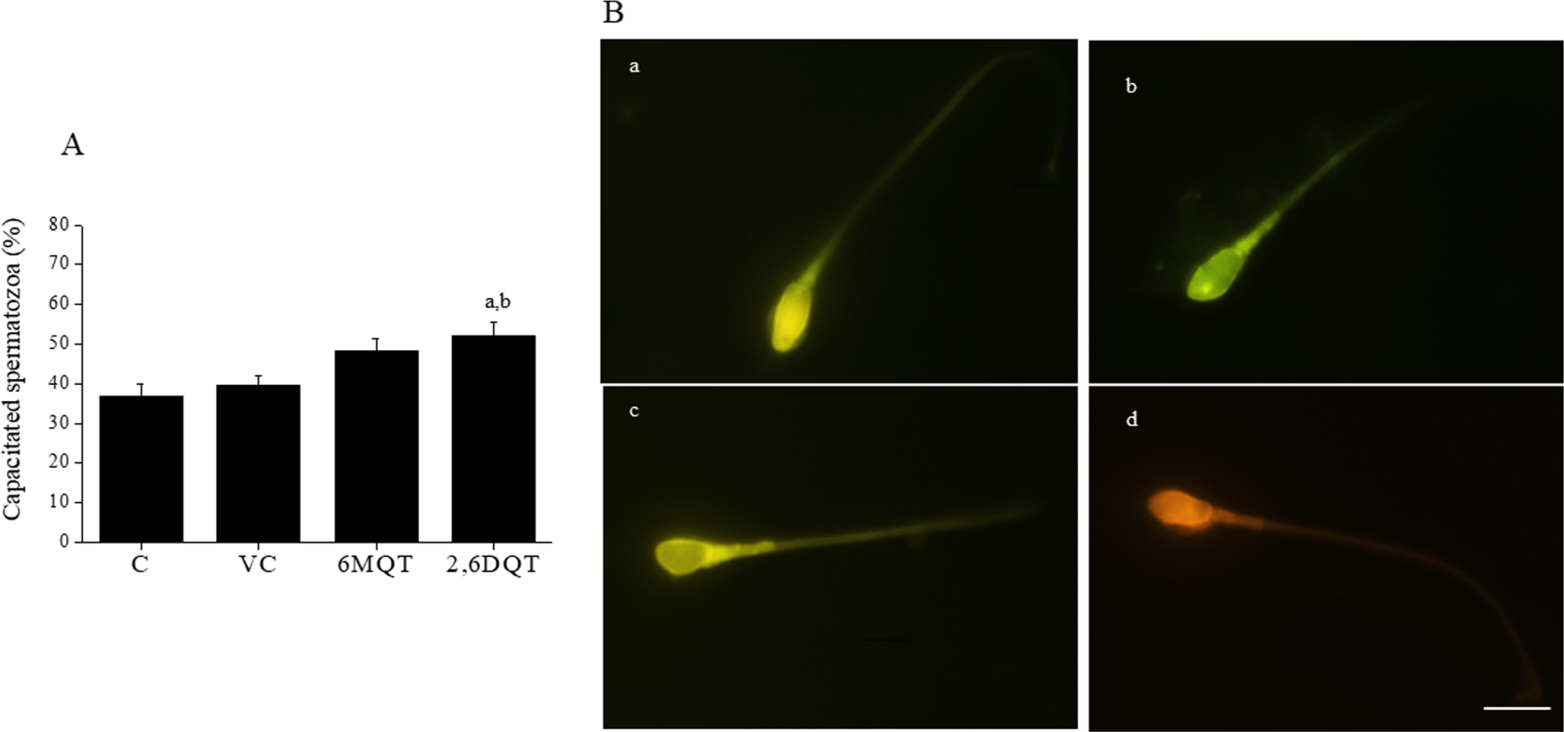
(A) Effect of 6MQT (0.05 μg/mL) and 2,6DQT (0.025 μg/mL) on, capacitation in in motile sperm fraction collected by swim-up at 1 h after incubation in vitro. (B) Representative images of (a) un-capacitated spermatozoa; (b) capacitated spermatozoa; (c) capacitated and acrosome reacted spermatozoa; and (d) dead spermatozoa, × 1000 magnification. Data represents mean ± SEM (*n* = 18). Scale bar represents 10 μm. ^a^*P* < 0.01 v/s C; ^b^*P* < 0.05 v/s VC

**Fig. 8:**
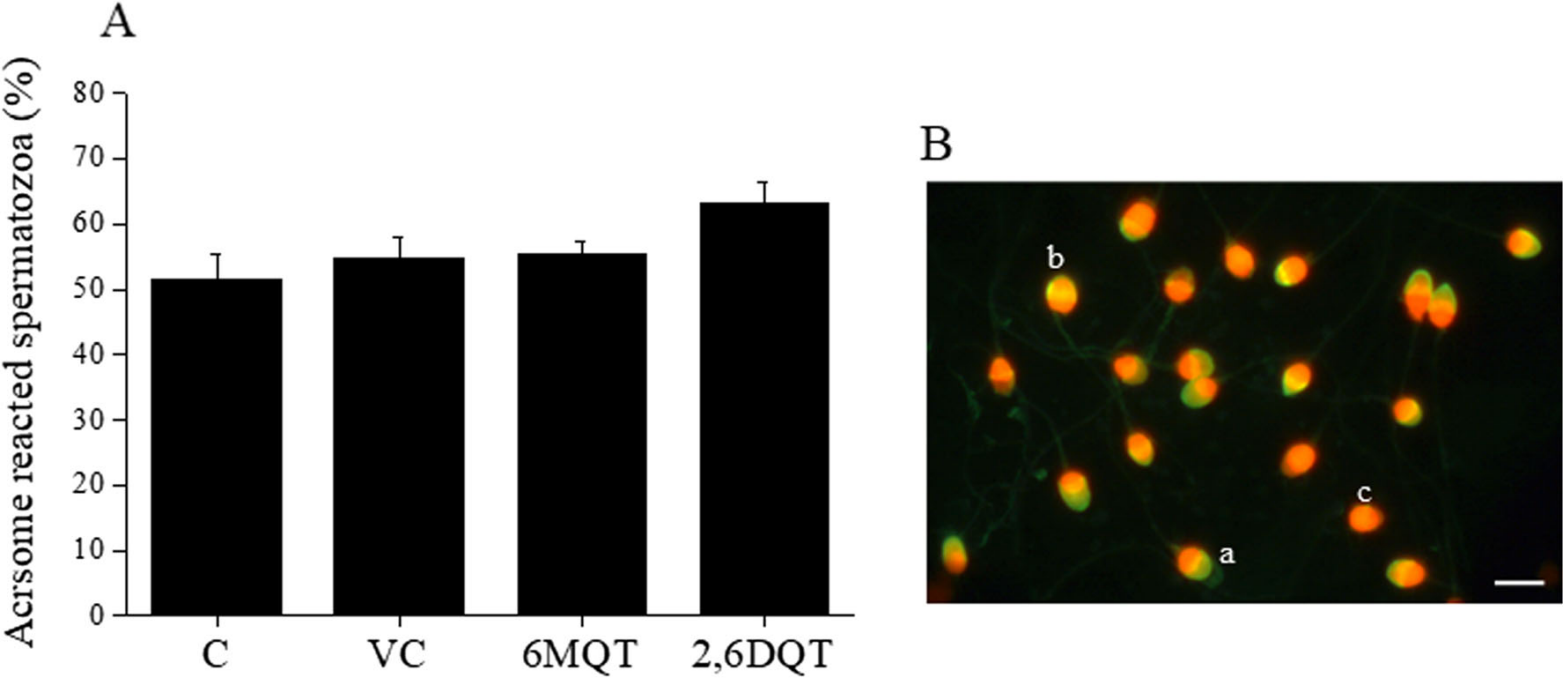
(A) Effect of 6MQT (0.05 μg/mL) and 2,6DQT (0.025 μg/mL) on ionophore-induced acrosome reaction in motile sperm fraction collected by swim-up at 1 h after incubation in vitro. (B) Representative images of (a) spermatozoa with intact acrosome; (b) spermatozoa with partially reacted acrosome; (c) spermatozoa undergone complete acrosome reaction, × 1000 magnification, (oil immersion). Data represents mean ± SEM (*n* = 18)

**Fig. 9:**
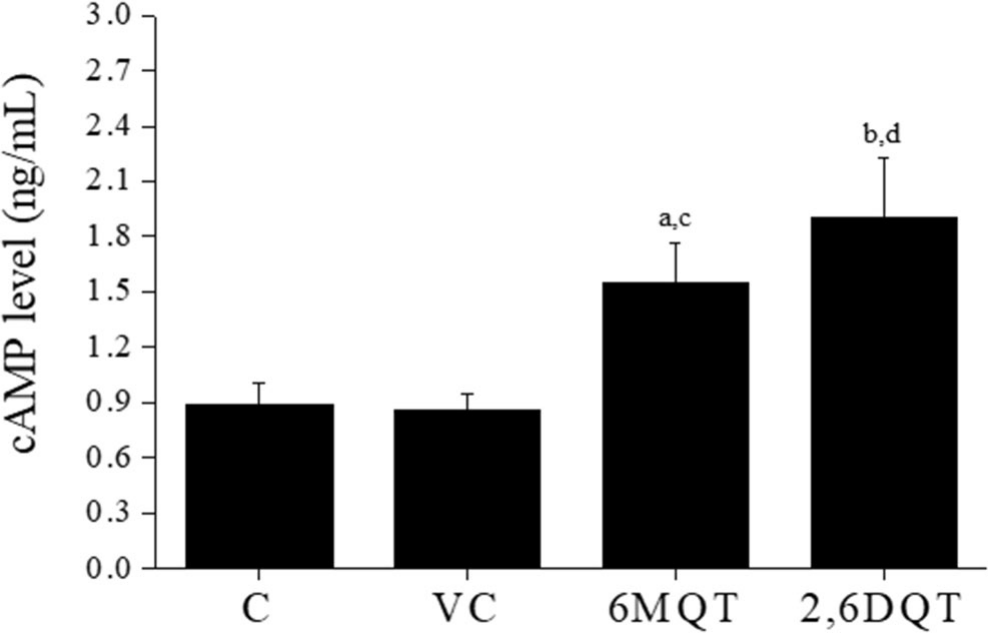
(A) Effect of 6MQT (0.05 μg/mL) and 2, 6DQT (0.025 μg/mL) on intracellular cAMP level in motile sperm fraction collected by swim-up at 1 h after incubation in vitro (*n* = 8). Data represents mean ± SEM. ^a^*P* < 0.05, ^b^*P* < 0.01 v/s C; ^c^*P* < 0.05, ^d^*P* < 0.01 v/s VC

Results of CTC assay at 1 h after incubation indicated that both 6MQT and 2,6DQT induced a significant increase in capacitation (*P* < 0.05) compared to control (Fig. 7). Similarly, at 1 h interval, the percentage of acrosome reacted spermatozoa was non-significantly higher in both 6MQT and 2,6DQT groups compared to control (Fig. 8). Among the two compounds, 2,6DQT group had higher percentage of capacitated (48.16 ± 3.09 vs 51.94 ± 3.56) and acrosome reacted spermatozoa compared to 6MQT group (63.1 ± 3.21 vs 55.5 ± 1.89). However, this difference was statistically not significant. Further, the intracellular cAMP level measured at 1 h after incubation was significantly higher in both 6MQT (*P* < 0.05) and 2,6DQT (*P* < 0.01) group compared to rest of the groups (Fig. 9).

### Tyrosine Phosphoprotein Level of Spermatozoa by Immunofluorescence

**Fig. 10:**
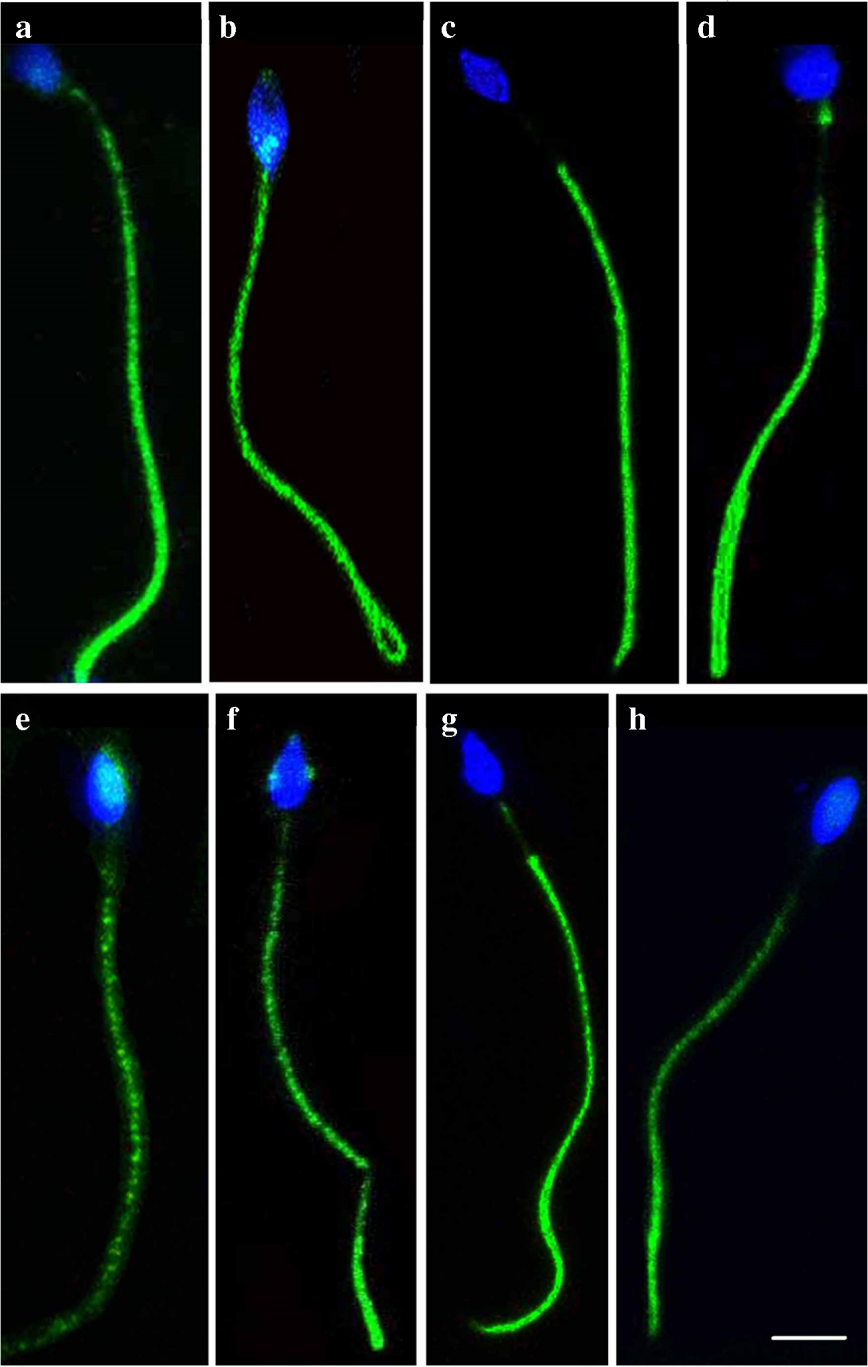
Confocal microscopic image of human spermatozoa depicting different patterns of localization of proteins phosphorylated at tyrosine residues in human spermatozoa processed with 6MQT (0.05 μg/mL) and 2, 6DQT (0.025 μg/mL) at 1 h after incubation in vitro (× 800 magnification). Spermatozoa were counterstained with DAPI. Scale bar represents 20 μm. (A), (B), and (G) Principal piece and midpiece (P + MP). (C) and (H) Principal piece (P). (D) Principal piece and neck (P + N). (E) Principal piece and acrosome region (P + A). (F) Principal piece and equatorial region (P + E)

**Fig. 11:**
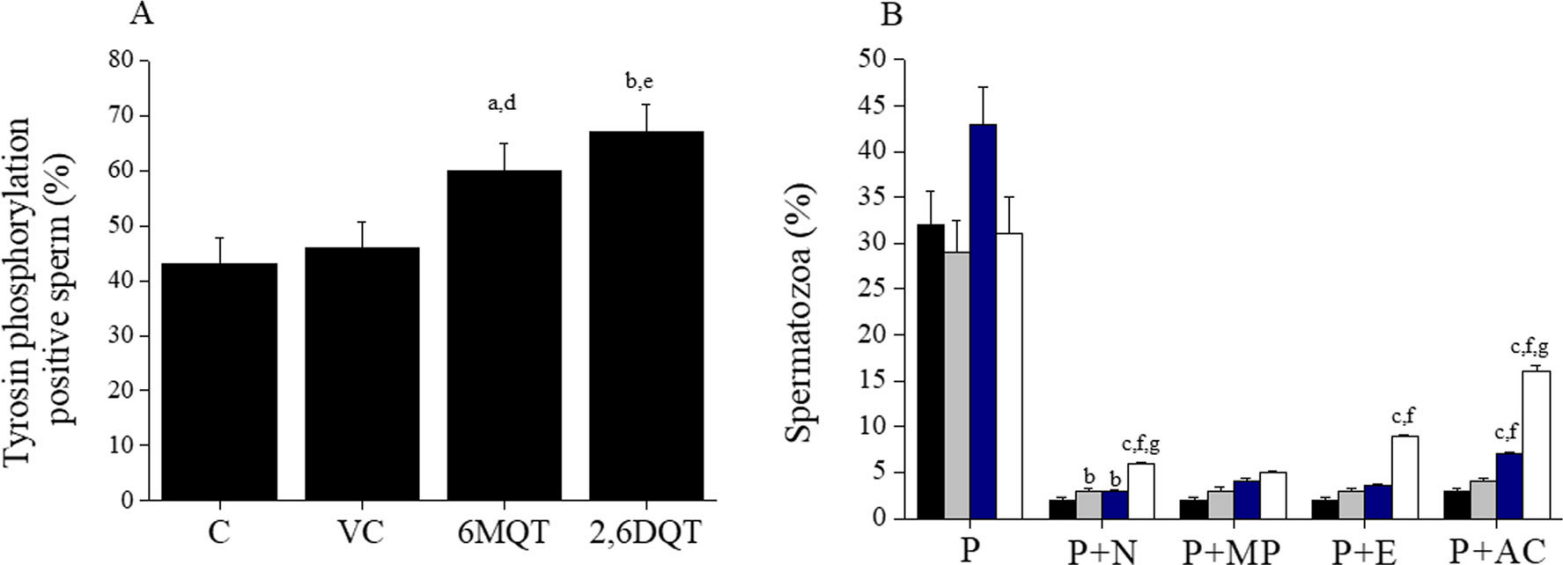
Effect of 6MQT (0.05 μg/mL) and 2, 6DQT (0.025 μg/mL) on tyrosine phosphorylation of sperm protein assessed by immunofluorescence in motile sperm fraction collected by swim-up at 1 h after incubation in vitro. (A) Percentage of tyrosine phosphorylated spermatozoa. (B) Classification of spermatozoa based on the localization in various parts of spermatozoa; P principle piece, MP midpiece, N neck region; E equatorial region, AC acrosomal cap region. Data represents mean ± SEM. (*n* = 8). ^a^*P* < 0.05, ^b^p < 0.01, ^c^*P* < 0.001 v/s C; ^d^*P* < 0.05, ^e^*P* < 0.01, ^f^*P* < 0.001 v/s VC; ^g^*P* < 0.001 v/s 6MQT. Control (C) (black bar); Vehicle control (VC) (gray bar); 6MQT (blue bar); 2,6DQT (white bar)

Immunolocalization of tyrosine phosphorylation in proteins of spermatozoa revealed that most predominant location was in the principal piece of the spermatozoa. However, expression in midpiece, end piece, and acrosomal cap was also observed (Fig. 10). Significantly higher percentage of spermatozoa with tyrosine phosphorylated proteins was observed in 6MQT (60.00 ± 4.84, *P* < 0.05) and 2,6DQT (67.00 ± 5.00, *P* < 0.01) group compared to control (43.00 ± 4.75) (Fig. 11(A)). Even though the total percentage of tyrosine phosphorylated spermatozoa were maximum in 2,6DQT group compared to rest of the groups, between two compounds, there was no significant difference. In addition, in 2,6DQT group, significantly higher percentage of spermatozoa (*P* < 0.001) had tyrosine phosphorylated proteins expressed in acrosomal cap (Fig. 11(B)).

### Analyzing Binding Affinity of 6MQT and 2,6DQT Compound for Various Isomers of Phosphodiesterase Enzymes

The affinity of these two compounds for PDE’s was compared with known inhibitors of specific PDEs such as pentoxifylline, IBMX, milrinone, rolipram, and sildenafil. Considering the C-score and Energy score, both the compounds (6MQT and 2,6DQT) bound to the predicted binding pockets of the proteins PDE5A, PDE1A, PDE7A, and PDE9A with more affinity than other proteins (PDE3B, PDE10A, and PDE4A) (Table 2).

**Table 2:** Computational docking of ligands (6MQT and 2,6DQT) to all major phosphodiesterases (PDEs). C-score, energy score, and predicted binding amino acid residues are indicated

Ligands	Protein	C-score*	Energy score	Predicted binding residues
Sildenafil	PDE5A	0.99	− 13.7	100,101,214,254,256,268,271,272,275,293,305,306,309
6MQT		0.99	− 11.2	100,101,214,254,256,268,271,272,275,293,305,306,309
2,6DQT		0.99	− 11.8	100,101,214,254,256,268,271,272,275,293,305,306,309
Pentoxifylline		0.99	− 9.0	100,101,214,254,256,268,271,272,275,293,305,306,309
(2-(Cyclopentylamino)thieno[3,2-d]pyrimidin-4(3H)-one derivative)	PDE7A	0.90	− 16.8	211,212,323,363,365,377,380,381,384,401,412,413,416
6MQT		0.90	− 12.1	211,212,323,363,365,377,380,381,384,401,412,413,416
2,6DQT		0.90	− 13.7	211,212,323,363,365,377,380,381,384,401,412,413,416
Pentoxifylline		0.90	− 10.5	211,212,323,363,365,377,380,381,384,401,412,413,416
**(**N~2~-(1-cyclopentyl-4-oxo-4,7-dihydro-1H-pyrazolo[3,4-d]pyrimidin-6-yl)-N-(4-methoxyphenyl)-D-alaninamide)	PDE9A	0.88	− 12.8	251,252,365,403,405,417,420,421,424,441,452,453,456
6MQT		0.88	− 12.5	251,252,365,403,405,417,420,421,424,441,452,453,456
2,6DQT		0.88	− 12.4	251,252,365,403,405,417,420,421,424,441,452,453,456
Pentoxifylline		0.88	− 9.8	251,252,365,403,405,417,420,421,424,441,452,453,456
8-MeIBMX	PDE1A	0.96	− 6.8	84,85,198,233,235,247,250,251,254,271,282,283,286
6MQT		0.96	− 11.1	84,85,198,233,235,247,250,251,254,271,282,283,286
2,6DQT		0.96	− 11.1	84,85,198,233,235,247,250,251,254,271,282,283,286
Pentoxifylline		0.96	− 9.0	84,85,198,233,235,247,250,251,254,271,282,283,286
Rolipram	PDE4A	0.48	− 11.3	432,433,546,592,594,606,609,610,613,630,641,642,645
6MQT		0.48	− 13.0	432,433,546,592,594,606,609,610,613,630,641,642,645
2,6DQT		0.48	− 12.9	432,433,546,592,594,606,609,610,613,630,641,642,645
Pentoxifylline		0.48	− 9.6	432,433,546,592,594,606,609,610,613,630,641,642,645
Papaverine	PDE10A	0.57	1.9	514,515,625,665,667,679,682,683,686,715,716,719
6MQT		0.57	4.9	514,515,625,665,667,679,682,683,686,715,716,719
2,6DQT		0.57	3.6	514,515,625,665,667,679,682,683,686,715,716,719
Pentoxifylline		Not done		
Milrinone	PDE3B	0.25	− 8.8	736,895,938,940,941,955,988,991
6MQT		0.25	− 12.6	736,895,938,940,941,955,988,991
2,6DQT		0.25	− 12.9	736,895,938,940,941,955,988,991
Pentoxifylline		0.25	− 10.6	736,895,938,940,941,955,988,991

*C-score is the confidence score of the prediction which ranges [0–1]. The higher C-score indicates better prediction. Lower energy score indicates better affinity of ligands towards protein binding pockets/sites

In this study, we have compared the energy score of the ligands 6MQT and 2,6DQT with the known inhibitors of PDEs. Based on the energy score, we have determined that the best binding affinity of the PDEs was found to be with the ligands 6MQT and 2,6DQT apart from their respective known inhibitors. We have selected the known inhibitors of the PDEs and allowed to dock with their respective PDEs first followed by the ligands, 6MQT and 2,6DQT, and pentoxifylline (a non-specific PDE inhibitor), and then on the basis of the binding energy, we have comprehended the binding affinity. We have found in case of PDE5A, the ligands 6MQT and 2,6DQT showed best energy score of (− 11.2, − 11.8) followed by pentoxifylline (− 9.0, a non-specific PDE inhibitor) and after the known inhibitor sildenafil (− 13.7). Likewise, in case of the other PDEs like PDE7A, PDE9A, and PDE1A, the energy score of 6MQT and 2,6DQT was better than the non-specific PDE inhibitor, pentoxifylline. In addition to that, the interacting amino acid residues of PDEs with the ligands 6MQT and 2,6DQT were identical with that of the known inhibitors.

### Effect of 6MQT and 2,6DQT on Fertilizing Ability of the Spermatozoa and Early Embryo Development

At 16 h post-insemination, the rate of fertilization in epididymal spermatozoa processed with 6MQT and 2,6DQT was 96.81 and 94.85% respectively, which was similar to control (96.28%) and vehicle control (94.62%). Percentage of zygotes progressed to 2 cell stage was also similar in 6MQT (96.7%) and 2,6DQT (95.65%) compared to control (92.27%) and vehicle control (91.45%). However, significantly lower blastocyst rate (*P* < 0.05) was observed in embryos derived from spermatozoa processed with 2,6DQT compared to rest of the groups indicating its adverse effects on early embryo development. On the other hand, the embryos derived from spermatozoa processed with 6MQT had similar blastocyst rate, with blastocysts having higher total cell number and significantly lower TUNEL index (*P* < 0.05) compared to control (Table 3).

**Table 3:** Effect of 6MQT (0.05 μg/mL) and 2,6DQT (0.025 μg/mL) on the fertilization and embryo developmental potential in vitro

Parameters	C	VC	6MQT	2,6DQT	Significance level
Number of oocytes inseminated	188	120	188	184	NA
Fertilization rate (%)	96.28	94.62	96.81	94.85	*P* = 0.902
2-cell rate (%)	92.27	91.45	96.7	95.65	*P* = 0.357
4-cell rate (%)	89.82	87.89	95.45	93.18	*P* = 0.124
Blastocyst rate (%)	86.82*	83.90	84.66	77.84*	*P* = 0.031
Hatching rate (%)	54.47	52.78	52.84	55.11	*p* = 0.876
Total cell number (mean ± SE)	85.62 ± 3.25	86.10 ± 3.67	92.18 ± 3.77	86.95 ± 3.22	*P* = 0.512
Apoptotic index (mean ± SE)	5.86 ± 0.94*	5.73 ± 0.96	3.25 ± 0.62*	2.00 ± 0.44	*P* = 0.02

*P < 0.05

## Discussion

In this study, we screened 9 novel quinoline derivatives for their beneficial effect on human sperm motility under in vitro conditions. Out of 9 compounds screened, 2 compounds (6MQT and 2,6DQT) exhibited significant beneficial effect on sperm motility enhancement. Both the compounds improved the sperm motility, and enhanced kinetic parameters such as VCL, VSL ALH, and VAP. While 6MQT and 2,6DQT had no significant difference in their effect on sperm functional competence, the developmental potential of embryos derived from sperm processed with 2,6DQT was poor.

Earlier studies have shown that the motility in spermatozoa can be modulated through various mechanisms. The most widely studied compounds are phosphodiesterase inhibitors. Xanthene derivatives like caffeine, theophylline, and pentoxifylline were shown to improve the sperm motility by inhibition of phosphodiesterase activity [32–34]. In this study, incubation of spermatozoa in media supplemented with 6MQT and 2,6DQT not only improved the in vitro sperm survival but also retained significantly higher percentage of progressive motility even at 24 h after incubation. Further, a significant increase in capacitation and improved kinetic parameters such as VSL, VCL, and ALH suggests its beneficial effect on sperm function. These physiological changes have been associated with elevated intracellular cAMP levels in spermatozoa, likely mediated by inhibition of phosphodiesterases (PDEs). Previous studies have reported high cAMP levels in cells treated with quinoline derivatives [35–37]. Further, the computational docking studies confirm a higher binding affinity of 6MQT and 2,6DQT to PDE subtypes- PDE1A, 3B, 4A, 5A, 7A, 9A, and 10A, compared to the commonly used sperm motility enhancer, pentoxifylline.

Increase in cAMP/cGMP level can in turn lead to tyrosine phosphorylation of sperm proteins [38], which is required for initiating hyperactivation of spermatozoa. In our study, significant increase in tyrosine phosphorylated sperm proteins was observed when they were processed in media containing 6MQT and 2,6DQT, indicating an increase in hyperactivated spermatozoa which also correlates with significant increase in VSL induced by these two compounds. However, a different pattern of tyrosine phosphorylation was observed in 2,6DQT, where majority of spermatozoa had phosphorylated proteins in acrosomal cap. This difference may be due to the onset of acrosome reaction since this group had non-significantly higher percentage of ionophore-induced acrosome reacted spermatozoa at 1 h after incubation compared to other groups.

Earlier studies have shown the presence of PDE subtypes in variety of mammalian tissues including human spermatozoa [39, 40]. It can be argued that selective small molecule inhibitors of spermatozoa specific PDE subtype are expected to have beneficial effect on the spermatozoa function without adverse effects. The docking results for 6MQT and 2,6DQT showed higher affinity for PDE5A, PDE1A, PDE7A, and PDE9A proteins. Among these, PDE1A plays an important role in the sperm function [39, 41]. While PDE5A, PDE7A, and PDE9A are known to contribute to the regulation of cAMP levels in mammalian tissues [42, 43], their role in sperm function has not yet been demonstrated. As with most research, this study also has a primary limitation since we have not evaluated the inhibitory effect of quinoline derivatives on human spermatozoa specific PDE subtypes by enzyme kinetic assays. Therefore, the experimental findings reported herein need to be interpreted with caution.

Although both quinoline derivatives (6MQT and 2,6DQT) exhibited similar motility enhancement properties, the mouse IVF results revealed that embryos derived from spermatozoa incubated with 6MQT displayed substantially improved blastocyst rates and quality compared to 2,6DQT, demonstrating its potential for future clinical applications. In this study, the spermatozoa were not washed off to remove trace amount of 6MQT and 2,6DQT prior to preparation of insemination droplet. Therefore, there is a risk of oocytes getting exposed to these compounds during fertilization. However, the improved fertilization and embryo development pattern observed in these two groups indicates that the presence of 6MQT and 2,6DQT during fertilization does not have any adverse consequence on the fertilization outcome. Since the mere enhancement of sperm motility does not ensure superior embryo development, it further highlights the need to test the developmental potential of embryos derived from spermatozoa processed with motility enhancement agents. Similar properties of sperm motility enhancers have been reported in earlier studies [15].

## Conclusions

To recapitulate, our study provides first-time evidence that a novel quinoline derivative, 6MQT, has significantly improved the sperm motility. The mouse IVF data show that 6MQT does not have adverse effects on the fertilization potential of spermatozoa and the developmental capacity of embryos derived from such sperm. However, further experiments are necessary to establish the clinical efficacy of 6MQT in IUI or IVF procedures and on the post-implantation developmental potential.

## Supplementary Information

ESM 1 (DOCX 31 kb).

## Data Availability

Will be provided on the request.

## References

[1] Van Voorhis BJ, Barnett M, Sparks AE, Syrop CH, Rosenthal G, Dawson J (2001). Effect of the total motile sperm count on the efficacy and cost-effectiveness of intrauterine insemination and in vitro fertilization. Fertil Steril.

[2] Hamilton JA, Cissen M, Brandes M, Smeenk JM, de Bruin JP, Kremer JA (2015). Total motile sperm count: a better indicator for the severity of male factor infertility than the WHO sperm classification system. Hum Reprod.

[3] Nagy ZP, Joris H, Verheyen G, Tournaye H, Devroey P, Van Steirteghem AC (1998). Correlation between motility of testicular spermatozoa, testicular histology and the outcome of intracytoplasmic sperm injection. Hum Reprod.

[4] Hammitt DG, Bedia E, Rogers PR, Syrop CH, Donovan JF, Williamson RA (1989). Comparison of motility stimulants for cryopreserved human semen. Fertil Steril.

[5] Lanzafame F, Chapman MG, Guglielmino A, Gearon CM, Forman RG (1994). Pharmacological stimulation of sperm motility. Hum Reprod.

[6] Barratt CLR, Bjorndahl L, De Jonge CJ, Lamb DJ, Osorio Martini F, McLachlan R (2017). The diagnosis of male infertility: an analysis of the evidence to support the development of global WHO guidance-challenges and future research opportunities. Hum Reprod Update.

[7] Dimitriadou F, Rizos D, Mantzavinos T, Arvaniti K, Voutsina K, Prapa A, Kanakas N (1995). The effect of pentoxifylline on sperm motility, oocyte fertilization, embryo quality, and pregnancy outcome in an in vitro fertilization program. Fertil Steril.

[8] Tasdemir I, Tasdemir M, Tavukcuoglu S (1998). Effect of pentoxifylline on immotile testicular spermatozoa. J Assist Reprod Genet.

[9] Tournaye H, Janssens R, Camus M, Staessen C, Devroey P, Van Steirteghem A (1993). Pentoxifylline is not useful in enhancing sperm function in cases with previous in vitro fertilization failure. Fertil Steril.

[10] Tournaye H, Janssens R, Devroey P, Van Steirteghem A (1994). The influence of pentoxifylline on motility and viability of spermatozoa from normozoospermic semen samples. Int J Androl.

[11] Yovich JM, Edirisinghe WR, Cummins JM, Yovich JL (1990). Influence of pentoxifylline in severe male factor infertility. Fertil Steril.

[12] Tasdemir M, Tasdemir I, Kodama H, Tanaka T (1993). Pentoxifylline-enhanced acrosome reaction correlates with fertilization in vitro. Hum Reprod.

[13] Imoedemhe DAG, Sigue AB, Pacpaco EA, Olazo AB (1992). Successful use of the sperm motility enhancer 2-deoxyadenosine in previously failed human in vitro fertilization. J Assist Reprod Genet.

[14] Lacham-Kaplan O, Trounson A (1993). The effects of the sperm motility activators 2-deoxyadenosine and pentoxifylline used for sperm micro-injection on mouse and human embryo development. Hum Reprod.

[15] Lacham-Kaplan O, Trounson A (1994). Embryo development capacity of oocytes fertilized by immature sperm and sperm treated with motility stimulants. Reprod Fertil Dev.

[16] Wiesner J, Ortmann R, Jomaa H, Schlitzer M (2003). New antimalarial drugs. Angew Chem Int Ed Eng.

[17] Shang XF, Morris-Natschke SL, Liu YQ, Guo X, Xu XS, Goto M, Li JC, Yang GZ, Lee KH (2018). Biologically active quinoline and quinazoline alkaloids part I. Med Res Rev.

[18] Kaufman TS, Ruveda EA (2005). The quest for quinine: those who won the battles and those who won the war. Angew Chem Int Ed Eng.

[19] Kouznetsov VV, Mendez LY, Gomez CM (2005). Recent progress in the synthesis of quinolones. Curr Org Chem.

[20] Shraddha MP, Kinjal DP, Rajesh HV, Shyamali NP, Hitesh DP (2014). Recent advances in the synthesis of quinolines: a review. RSC Adv.

[21] Bawa S, Kumar S, Drabu S, Kumar R (2010). Structural modifications of quinoline-based antimalarial agents: recent developments. J Pharm Bioallied Sci.

[22] Ozyanik M, Demirci S, Bektas H, Demirbas N, Demirbas A, Karaoglu SA (2012). Preparation and antimicrobial activity evaluation of some quinoline derivatives containing an azole nucleus. Turk J Chem.

[23] Graves PR, Kwiek JJ, Fadden P, Ray R, Hardeman K, Coley AM, Foley M, Haystead TAJ (2002). Discovery of novel targets of quinoline drugs in the human purine binding proteome. Mol Pharmacol.

[24] Desai U, Mitragotri SD, Thopate TS, Pore DM, Wadgaonkar PP (2006). A highly efficient synthesis of trisubstituted quinolines using sodium hydrogensulfate on silica gel as a reusable catalyst. ARKIVOC.

[25] Hamaguchi W, Masuda N, Isomura M, Miyamoto S, Kikuchi S, Amano Y, Honbou K, Mihara T, Watanabe T (2013). Design and synthesis of novel benzimidazole derivatives as phosphodiesterase 10A inhibitors with reduced CYP1A2 inhibition. Bioorg Med Chem.

[26] Somagond SM, Kamble RR, Kattimani PP, Shaikh SKJ, Dixit SR, Joshi SD, Devarajegowda HC (2018). Design, docking, and synthesis of quinoline-2H-1,2,4-triazol-3(4H)-ones as potent anticancer and antitubercular agents. ChemistrySelect..

[27] Salian SR, Nayak G, Kumari S, Patel S, Gowda S, Shenoy Y, Sugunan S, G.K R, Managuli RS, Mutalik S, Dahiya V, Pal S, Adiga SK, Kalthur G (2019). Supplementation of biotin to sperm preparation medium enhances fertilizing ability of spermatozoa and improves preimplantation embryo development. J Assist Reprod Genet.

[28] Kotdawala AP, Kumar S, Salian SR, Thankachan P, Govindraj K, Kumar P, Kalthur G, Adiga SK (2012). Addition of zinc to human ejaculate prior to cryopreservation prevents freeze-thaw-induced DNA damage and preserves sperm function. J Assist Reprod Genet.

[29] Isaac AV, Kumari S, Nair R, Urs DR, Salian SR, Kalthur G, Adiga SK, Manikkath J, Mutalik S, Sachdev D, Pasricha R (2017). Supplementing zinc oxide nanoparticles to cryopreservation medium minimizes the freeze-thaw-induced damage to spermatozoa. Biochem Biophys Res Commun.

[30] Kalthur G, Salian SR, Nair R, Mathew J, Adiga SK, Kalthur SG, Zeegers D, Hande MP (2016). Distribution pattern of cytoplasmic organelles, spindle integrity, oxidative stress, octamer-binding transcription factor 4 (Oct4) expression and developmental potential of oocytes following multiple superovulation. Reprod Fertil Dev.

[31] Wu Q, Peng Z, Zhang Y, Yang J (2018). COACH-D: improved protein-ligand binding sites prediction with refined ligand-binding poses through molecular docking. Nucleic Acids Res.

[32] Jiang CS, Kilfeather SA, Pearson RM, Turner P (1984). The stimulatory effects of caffeine, theophylline, lysine-theophylline and 3-isobutyl-1-methylxanthine on human sperm motility. Br J Clin Pharmacol.

[33] Levin RM, Greenberg SH, Wein AJ (1981). Quantitative analysis of the effects of caffeine on sperm motility and cyclic adenosine 3′,5′-monophosphate (AMP) phosphodiesterase. Fertil Steril.

[34] Loughlin KR, Agarwal A (1992). Use of theophylline to enhance sperm function. Arch Androl.

[35] Ukita T, Sugahara M, Terakawa Y, Kuroda T, Wada K, Nakata A, Ohmachi Y, Kikkawa H, Ikezawa K, Naito K (1999). Novel, potent, and selective phosphodiesterase-4 inhibitors as antiasthmatic agents: synthesis and biological activities of a series of 1-pyridylnaphthalene derivatives. J Med Chem.

[36] Bi Y, Stoy P, Adam L, He B, Krupinski J, Normandin D, Pongrac R, Seliger L, Watson A, Macor JE (2004). Quinolines as extremely potent and selective PDE5 inhibitors as potential agents for treatment of erectile dysfunction. Bioorg Med Chem Lett.

[37] Fiorito J, Saeed F, Zhang H, Staniszewski A, Feng Y, Francis YI, Rao S, Thakkar DM, Deng SX, Landry DW, Arancio O (2013). Synthesis of quinoline derivatives: discovery of a potent and selective phosphodiesterase 5 inhibitor for the treatment of Alzheimer’s disease. Eur J Med Chem.

[38] Aitken RJ, Harkiss D, Knox W, Paterson M, Irvine DS (1998). A novel signal transduction cascade in capacitating human spermatozoa characterised by a redox-regulated, cAMP-mediated induction of tyrosine phosphorylation. J Cell Sci.

[39] Beavo JA (1995). Cyclic nucleotide phosphodiesterases: functional implications of multiple isoforms. Physiol Rev.

[40] Conti M, Nemoz G, Sette C, Vicin E (1995). Recent progress in understanding the hormonal regulation of phosphodiesterases. Endocr Rev.

[41] Lefievre L, de Lamirande E, Gagnon C (2002). Presence of cyclic nucleotide phosphodiesterases PDE1A, existing as a stable complex with calmodulin, and PDE3A in human spermatozoa. Biol Reprod.

[42] Lee R, Wolda S, Moon E, Esselstyn J, Hertel C, Lerner A (2002). PDE7A is expressed in human B-lymphocytes and is up-regulated by elevation of intracellular cAMP. Cell Signal.

[43] Keravis T, Lugnier C (2012). Cyclic nucleotide phosphodiesterase (PDE) isozymes as targets of the intracellular signalling network: benefits of PDE inhibitors in various diseases and perspectives for future therapeutic developments. Br J Pharmacol.

