# Supplementary material for: Quinoline Derivative Enhances Human Sperm Motility and Improves the Functional Competence
Source: Reprod Sci. 2020 Nov 25;28(5):1316–32. doi: 10.1007/s43032-020-00382-5 (PMC8076127; doi:10.1007/s43032-020-00382-5)
Supplement: Supplementary file 1 — (DOCX 31 kb). [file 43032_2020_382_MOESM1_ESM.docx]

**Novel quinoline derivative enhances human sperm motility and improves the functional competence**

**Table1: Demographic characteristics and semen parameters in normozoospermic men recruited for the study (**n=73)

| **Parameters** | **Mean ± SEM** |
| --- | --- |
| Age (years) | 36.26 ± 0.58 |
| Sperm concentration (millions/mL) | 61.78 ± 5.71 |
| Total motility (%) | 62.05 ± 1.81 |
| Progressive motility (%) | 45.61 ± 2.05 |
| Morphology (%) | 30.95 ± 5.06 |
| Viability (%) | 64.62 ± 2.76 |

*Screening of quinoline derivatives for sperm motility enhancement*

After routine semen analysis, the normozoospermic samples (n=18) were used for screening the beneficial effect of newly synthesized 9 quinoline derivatives on human sperm motility under *in vitro* conditions. For this, equal volume of liquefied human semen samples was divided into several parts and washed with EBSS medium by centrifuging at 1800 rpm for 8 min. The pellet obtained was overlaid with EBSS medium containing various concentrations of quinoline derivatives (0.5 – 5 µg/mL) and incubated for 1 h in an incubator maintained at 37 °C, 5% CO_2_. The motile spermatozoa were collected from the overlay without disturbing the pellet and further incubated up to 4 h at 37 °C, 5% CO_2_. For motility assessment (1 h and 4 h), 10 µL of sperm suspension was placed on a microscopic slide on which coverslip was placed. Based on the motility pattern spermatozoa were categorized into progressive, non-progressive and immotile spermatozoa in 200 spermatozoa (WHO, 2010) by observing under the light microscope (400x magnification).

Among the 9 quinoline derivatives screened, 2 compounds - 6MQT ((4-((2-Chloro-6-methoxyquinolin-3-yl)methyl)-2-(4-methoxyphenyl)-2*H*-1,2,4-triazol-3(4*H*)-one) and 2,6DQT ((4-((2,6-Dichloroquinolin-3-yl)methyl)-2-(4-methoxyphenyl)-2*H*-1,2,4-triazol-3(4*H*)-one) showed increase in total and progressive motility compared to control and other quinoline derivatives (Table 2) at 4 h interval at 0.5 µg/mL concentration. These two lead compounds were further screened at concentrations ranging from 0.25 to 0.025 µg/mL to elucidate the optimum concentration (Table 3 and 4) for motility enhancement by incubation of swim up motile sperm fraction further up to 48 h at 37 °C, 5% CO_2_. Based on the total and progressive motility pattern at 24 and 48 h, the optimum concentration for 6MQT and 2,6DQT was found to be 0.05 µg/mL and 0.025 µg/mL respectively (Table 4). Therefore, for further studies on assessing the sperm functional characteristics, these two concentrations were used.

**Table 2: Screening of 9 quinoline derivatives (concentrations from 0.025 - 1 μg/mL) for sperm motility enhancement in human spermatozoa at 1 h and 4 h interval**

|  | \| Groups \| \| --- \| | Conc.  (µg/mL) | \| Motility (1 h) \| \| --- \| | | \| Motility (4 h) \| \| --- \| | |
| --- | --- | --- | --- | --- | --- | --- | --- | --- | --- |
|  |  |  | Total motility (%) | Progressive motility (%) | Total motility (%) | Progressive motility (%) |
| \| 1st set  (n=3) \| \| --- \| | Control | 0 | 81.00 ± 7.57 | 63.00 ± 4.63 | 59.33 ± 4.33 | 46.00 ± 2.51 |
|  | 2CQT | 0.5 | 82.67 ± 3.71 | 56.60 ± 3.33 | 71.67 ± 8.95 | 45.67 ± 8.45 |
|  |  | 1 | 83.33 ± 5.81 | 57.33 ± 2.33 | 63.67 ± 7.67 | 49.67 ± 7.67 |
|  |  | 5 | 71.67 ± 5.46 | 42.67 ± 6.44 | 55.60 ± 3.18 *^a^* | 33.67 ± 5.46 *^b, c^* |
|  | 6MQT | 0.5 | 89.67 ± 5.49 | 74.33 ± 4.33 | 86.00 ± 4.36 | 67.00 ± 3.61 |
|  |  | 1 | 78.33 ± 3.38 | 60.00 ± 6.11 | 75.67 ± 0.88 | 58.00 ± 2.65 |
|  |  | 5 | 77.00 ± 6.35 | 58.33 ± 7.17 | 64.00 ± 1.53 | 47.00 ± 3.48 |
|  | 2HQT | 0.5 | 77.67 ± 7.88 | 59.67 ± 6.39 | 67.00 ± 2.00 | 50.00 ± 3.22 |
|  |  | 1 | 78.00 ± 4.16 | 58.67 ± 3.71 | 69.67 ± 8.51 | 53.33 ± 4.33 |
|  |  | 5 | 81.00 ± 4.51 | 58.60 ± 2.03 | 66.33 ± 5.36 | 51.00 ± 3.00 |
|  | 2PQT | 0.5 | 78.67 ± 5.78 | 60.00 ± 2.31 | 63.33 ± 4.67 | 50.00 ± 3.06 |
|  |  | 1 | 80.33 ± 4.18 | 61.00 ± 3.51 | 60.67 ± 3.84 | 47.67 ± 4.33 |
|  |  | 5 | 76.67 ± 6.89 | 55.33 ± 6.17 | 56.57 ± 5.78 *^a^* | 41.67 ± 4.63 *^a^* |
| \| 2nd set  (n=3) \| \| --- \| | Control | 0 | 93.00 ± 2.00 | 57.30 ± 5.80 | 94.30 ± 4.70 | 60.00 ± 5.70 |
|  | 2,6CQT | 0.5 | 92.30 ± 0.70 | 53.70 ± 11.9 | 95.30 ± 2.00 | 66.30 ± 7.30 |
|  |  | 1 | 95.00 ± 1.20 | 63.30 ± 4.10 | 89.30 ± 5.50 | 53.30 ± 7.70 |
|  |  | 5 | 87.70 ± 2.40 | 40.00 ± 12.7 | 92.70 ± 3.30 | 68.70 ± 5.40 |
|  | 2,6DQT | 0.5 | 94.63 ± 0.75 | 73.60 ± 3.77 | 93.33 ± 2.26 | 78.00 ± 4.28 |
|  |  | 1 | 93.75 ± 1.77 | 63.25 ± 7.83 | 87.83 ± 3.17 | 65.67 ± 4.40 |
|  |  | 5 | 89.88 ± 1.85 | 60.25 ± 6.83 | 89.00 ± 3.56 | 66.00 ± 5.32 |
| \| 3rd set  (n=3) \| \| --- \| | Control | 0 | 88.00 ± 1.70 | 71.30 ± 6.70 | 82.30 ± 5.40 | 70.70 ± 6.30 |
|  | 2,OQT | 0.5 | 88.70 ± 0.30 | 73.00 ± 5.90 | 89.70 ± 4.80 | 78.00 ± 4.00 |
|  |  | 1 | 83.00 ± 4.50 | 66.30 ± 4.70 | 78.00 ± 5.80 | 65.70 ± 3.80 |
|  |  | 5 | 82.30 ± 3.80 | 68.30 ± 4.10 | 80.00 ± 6.10 | 66.70 ± 7.20 |
|  | 2,6MQT | 0.5 | 82.00 ± 3.20 | 64.00 ± 3.00 | 81.00 ± 6.00 | 71.30 ± 4.80 |
|  |  | 1 | 80.70 ± 2.70 | 63.30 ± 3.30 | 88.00 ± 4.40 | 76.70 ± 3.30 |
|  |  | 5 | 86.30 ± 2.20 | 68.00 ± 4.50 | 88.30 ± 1.80 | 75.30 ± 2.40 |
|  | 2,6HQT | 0.5 | 85.00 ± 4.40 | 68.00 ± 6.00 | 84.30 ± 4.90 | 71.00 ± 4.60 |
|  |  | 1 | 83.00 ± 3.20 | 65.70 ± 3.50 | 81.30 ± 4.90 | 69.30 ± 3.50 |
|  |  | 5 | 83.30 ± 4.40 | 64.00 ± 2.10 | 79.00 ± 4.50 | 68.00 ± 3.60 |

^a^*P* < 0.05, ^b^*P* < 0.01 v/s 6MQT 0.5 µg/mL; ^c^*P* < 0.05 v/s 6MQT 1 µg/mL

**ble 3: Screening of 6MQT, 2,6DQT and 2,6CQT (concentration of 0.25, 0.1, and 0.05 µg/mL) for sperm motility enhancement in human spermatozoa at 1, 24 and 48 h interval**

| 4^th^ set  (n= 6) | Concentration (µg/mL) | Motility (1 h) | | Motility (24 h) | | Motility (48 h) | |
| --- | --- | --- | --- | --- | --- | --- | --- |
| Quinoline derivatives |  | Total (%) | Progressive (%) | Total (%) | Progressive (%) | Total (%) | Progressive (%) |
| Control | 0.0 | 73.80 ± 7.60 | 53.00 ± 8.40 | 57.80 ± 9.40 | 40.50 ± 7.60 | 22.7 ± 4.30 | 9.70 ± 4.20 |
| 6MQT | 0.05 | 74.00 ± 5.60 | 54.00 ± 7.50 | 61.70 ± 8.00 | 43.70 ± 7.40 | 25.50 ± 4.00 | 12.0 ± 5.10 |
|  | 0.1 | 73.80 ± 5.80 | 53.30 ± 7.80 | 57.30 ± 10.8 | 38.80 ± 8.90 | 21.70 ± 3.00 | 9.20 ± 3.00 |
|  | 0.25 | 71.20 ± 7.30 | 52.50 ± 8.80 | 58.20 ± 10.9 | 41.00 ± 9.60 | 21.20 ± 4.40 | 8.30 ± 4.20 |
| 2,6DQT | 0.05 | 74.50 ± 6.70 | 55.20 ± 6.10 | 60.70 ± 9.80 | 41.30 ± 8.80 | 21.50 ± 3.80 | 8.80 ± 3.70 |
|  | 0.1 | 78.70 ± 6.10 | 58.70 ± 5.40 | 63.30 ± 9.60 | 45.80 ± 9.10 | 22.20 ± 2.10 | 8.20 ± 3.10 |
|  | 0.25 | 76.50 ± 7.20 | 56.00 ± 6.70 | 63.00 ± 11.1 | 44.50 ± 9.70 | 22.70 ± 4.10 | 9.00 ± 3.80 |

**Table 4: Screening of 6MQT and 2,6DQT (concentration of 0.1, 0.05, 0.025 µg/mL) for sperm motility enhancement in human spermatozoa at 1, 24 and 48 h interval**

| 5^th^ Set  (n= 3)  Groups | Concentration (µg/mL) | Motility (1 h) | | Motility (24 h) | | Motility (48 h) | |
| --- | --- | --- | --- | --- | --- | --- | --- |
|  |  | Total (%) | Progressive (%) | Total (%) | Progressive (%) | Total (%) | Progressive (%) |
| Control | 0.0 | 85.70 ± 6.00 | 56.70 ± 13.70 | 86.30 ± 5.70 | 78.70 ± 9.30 | 55.00 ± 4.20 | 44.00 ± 4.00 |
| 6MQT | 0.025 | 78.70 ± 7.30 | 60.70 ± 14.00 | 67.30 ± 20.2 | 52.70 ± 18.9 | 46.30 ± 17.5 | 34.30 ± 15.9 |
|  | 0.05 | 81.70 ± 8.00 | 67.00 ± 15.80 | 79.30 ± 4.10 | 71.30 ± 9.40 | 71.30 ± 5.20 | 57.30 ± 1.50 |
|  | 0.1 | 86.70 ± 0.90 | 71.00 ± 12.10 | 80.70 ± 3.80 | 64.30 ± 8.80 | 65.30 ± 2.60 | 52.30 ± 1.90 |
| 2,6DQT | 0.025 | 85.70 ± 2.30 | 68.00 ± 6.70 | 90.00 ± 1.00 | 76.00 ± 7.10 | 66.00 ± 10.4 | 52.30 ± 5.50 |
|  | 0.05 | 88.00 ± 8.20 | 71.70 ± 15.40 | 90.30 ± 1.50 | 78.30 ± 6.20 | 61.00 ± 6.00 | 48.70 ± 2.60 |
|  | 0.1 | 87.30 ± 2.60 | 68.00 ± 9.20 | 86.00 ± 0.60 | 77.30 ± 6.20 | 56.30 ± 3.90 | 45.00 ± 1.70 |
